# Structural and Functional Characterization of a Unique AP Endonuclease From *Deinococcus radiodurans*

**DOI:** 10.3389/fmicb.2020.01178

**Published:** 2020-06-05

**Authors:** Yuan He, Yiyi Wang, Chen Qin, Ying Xu, Kaiying Cheng, Hong Xu, Bing Tian, Ye Zhao, Liangyan Wang, Yuejin Hua

**Affiliations:** MOE Key Laboratory of Biosystems Homeostasis and Protection, College of Life Sciences, Institute of Biophysics, Zhejiang University, Hangzhou, China

**Keywords:** *Deinococcus radiodurans*, base excision repair, AP endonuclease, DrXth, genome stability

## Abstract

Various endogenous and exogenous agents cause DNA damage, including apurinic/apyrimidinic (AP) sites. Due to their cytotoxic effects, AP sites are usually cleaved by AP endonuclease through the base excision repair (BER) pathway. *Deinococcus radiodurans*, an extraordinary radiation-resistant bacterium, is known as an ideal model organism for elucidating DNA repair processes. Here, we have investigated a unique AP endonuclease (DrXth) from *D. radiodurans* and found that it possesses AP endonuclease, 3′-phosphodiesterase, 3′-phosphatase, and 3′–5′ exonuclease but has no nucleotide incision repair (NIR) activity. We also found that Mg^2+^ and Mn^2+^ were the preferred divalent metals for endonuclease and exonuclease activities, respectively. In addition, DrXth were crystallized and the crystals diffracted to 1.5 Å. Structural and biochemical analyses demonstrated that residue Gly198 is the key residue involved in the substrate DNA binding and cleavage. Deletion of the *drxth* gene in *D. radiodurans* caused elevated sensitivity to DNA damage agents and increased spontaneous mutation frequency. Overall, our results indicate that DrXth is an important AP endonuclease involved in BER pathway and functions in conjunction with other DNA repair enzymes to maintain the genome stability.

## Introduction

Genomic DNA regularly suffers accidental damage caused by endogenous and exogenous agents. One of the most abundant types of damage is apurinic/apyrimidinic (AP) sites, resulting from spontaneous water-mediated depurination or depyrimidination, or hydrolysis of the *N*-glycosyl bond between the base and the sugar-phosphate backbone by DNA glycosylase (Wilson and Barsky, 2001). Under physiological conditions, ~9,000 AP sites are generated in a typical cell per day (Nakamura et al., 1998). As frequent intermediates, AP sites are cytotoxic and increase mutagenesis through deletions, insertions, or substitutions if bypassed by DNA polymerases (Loeb and Preston, 1986). The major contribution to AP sites cleavage is provided by AP endonuclease, a crucial enzyme in the base excision repair (BER) pathway (Wilson and Barsky, 2001). AP endonuclease incises DNA on the 5′ side of the abasic site, leaving a 3′-terminal hydroxyl (3′-OH) and 5′-deoxyribose phosphate (dRP) following hydrolytic reaction (Dyrkheeva et al., 2016). Other AP sites are cleaved on the 3′ side by bifunctional DNA glycosylase *via* an AP lyase mechanism, leaving a 5′-phosphate and a 3′-α, β-4-hydroxypenten-2-al (3′-PUA) (McCullough et al., 2001; Sahan et al., 2018). In either situation, the blocks are subsequently removed by corresponding enzymes to allow polymerases to fill the gap, and nicks are subsequently sealed by DNA ligase (Morita et al., 2010). Alternatively, the nucleotide incision repair (NIR) pathway provides a back-up repair pathway that operates alongside the classical BER pathway (Ishchenko et al., 2003). For instance, APE1 can incise DNA on the 5′ side of oxidatively damaged bases in a DNA glycosylase-independent manner, leaving 3′-hydroxyl (3′-OH) and 5′-phosphate (5′-P) termini (Ischenko and Saparbaev, 2002).

Based on structural and sequence similarities, all known AP endonucleases can be categorized into two families: *Escherichia coli* exonuclease III (e.g., Xth, Mg^2+^-dependent) and endonuclease IV (e.g., Nfo, independent of external divalent cations) (Mol et al., 2000a). In *E. coli*, exonuclease III accounts for 90% of AP endonuclease activity (Weiss, 1976). In *Saccharomyces cerevisiae*, Apn1 and Apn2 are EndoIV and ExoIII homologs, respectively, that function in alternate pathways for the repair of AP sites (Demple et al., 1997; Unk et al., 2000). Human cells only contain two ExoIII family AP endonucleases (APE1 and APE2). APE1 is a multifunctional DNA repair enzyme that possesses AP endonuclease, 3′-phosphodiesterase, 3′-phosphatase, 3′–5′-exonuclease, and NIR enzymatic activities, and the endonuclease activity of APE2 is distinctly lower than that of APE1 (Dyrkheeva et al., 2016; Whitaker and Freudenthal, 2018).

Currently, several crystal structures of ExoIII family AP endonuclease have been solved in eukaryote, prokaryote, and archaea (Mol et al., 1995; Gorman et al., 1997; Schmiedel et al., 2009). APE1-DNA complex structure provides mechanistic details for elucidating the AP endonuclease catalytic reaction (Mol et al., 2000b). A metal cofactor is necessary for the phospho-diester hydrolysis reaction of incision to the DNA backbone, positioning a nucleophilic water molecule and neutralizing the negative charge to stabilize the transition state (Beernink et al., 2001; Tsutakawa et al., 2013). Magnesium is required for both endonuclease and exonuclease activity (Chou and Cheng, 2003). Key catalytic amino acid residues such as Asp210 and Asn212 in APE1 coordinate the metal ion and nucleophilic water to facilitate attack on the abasic site (Freudenthal et al., 2015; Batebi et al., 2018).

*Deinococcus radiodurans* belongs to the *Deinococcus-Thermus* phylum with extraordinary resistance to a variety of extreme environment and agents including desiccation, ionizing radiation (IR), ultraviolet (UV) radiation, oxidative stress, and toxic chemicals (Battista, 1997; Munteanu et al., 2015). Although the BER pathway is highly conserved in prokaryotes and eukaryotes, this DNA repair pathway has some specific features in *D. radiodurans*. For instance, this organism encodes a relatively large number of DNA glycosylases and contains three bifunctional endonuclease III proteins, unlike most other species (Timmins and Moe, 2016). Compared with *E. coli*, mismatch-specific uracil DNA glycosylases MUG (DR_0715) and AlkA (DR_2584) expand the substrate specificity (Moe et al., 2006, 2012). Previous research indicates that DrEndoIII1 (DR_2438) and DrEndoIII2 (DR_0289) possess AP-lyase activity (Sarre et al., 2015). In addition, whole-genome sequencing has discovered only one AP endonuclease in *D. radiodurans*, whose structure and function have been little studied in extremophiles (Makarova et al., 2001).

In the present study, we showed that DR_0354 protein (DrXth), the exonuclease III homolog, possesses AP endonuclease, 3′-phosphodiesterase, 3′-phosphatase, and 3′–5′-exonuclease activities. The endonuclease and exonuclease activities of DrXth prefer Mg^2+^ and Mn^2+^, respectively. The three-dimensional structure of DrXth is determined to 1.5 Å resolution. Furthermore, structural and biochemical analyses suggest that Gly198 is a key residue for binding the DNA substrate. In addition, deletion of the *drxth* gene led to increased sensitivity to stress-induced DNA damage and a highly elevated spontaneous mutation frequency.

## Materials and Methods

### Plasmids and Bacterial Strains

All plasmids, bacterial strains, and primers used in this study are listed in Supplementary Tables S1 and S2. *D. radiodurans* strains were grown in TGY medium (0.5% tryptone, 0.1% glucose, and 0.3% yeast extract) or on TGY plate (TGY medium supplemented with 1.5% agar) at 30°C. *E. coli* strains were grown in LB medium (1% tryptone, 0.5% yeast extract, and 1% sodium chloride) or on LB plate (LB medium supplemented with 1.5% agar) at 37°C. Antibiotics were appropriately added into TGY medium (20 mg/L kanamycin, 4 mg/L chloramphenicol, 10 mg/L streptomycin, and 50 mg/L rifampicin) and LB medium (40 mg/L kanamycin and 100 mg/L ampicillin) in different situations.

### Gene Disruption and Complementation

Tripartite ligation method used to disrupt *D. radiodurans* genes was performed as described previously with some modification (Wang et al., 2012). Briefly, the upstream and downstream fragments of the target gene were amplified by primers that have *Bam*HI and *Hind*III restriction enzyme sites, respectively (Supplementary Table S2). After digestion, the fragments were ligated to a streptomycin resistance gene that was digested with *Bam*HI and *Hind*III previously. The tripartite ligation product was transformed into *D. radiodurans*. The mutants were selected on the TGY plate with relevant streptomycin. For complementation, the target gene was amplified by primers with *Nde*I and *Bam*HI (Supplementary Table S2). After digestion, the fragment was inserted into a shuttle vector pRADK, which has the same restriction enzyme sites. Then, the recombinant plasmid was transformed into mutant strains.

### Protein Expression and Purification

Full length *drxth* gene (used for biochemical analysis), N-terminal truncated *Δ22 drxth* gene (used for crystallization), full length *APE1* gene, and *drpolA-C* gene were amplified by PCR and cloned into expression vector pET28a at *Nde*I and *Bam*HI site (Supplementary Tables S1 and S2). The encoded proteins carry an N-terminal 6× His-tag sequence. For His-Strep-DrXth, *drxth* gene was ligated with the pET28S expression vector to facilitate co-expression of His-tag with a Strep-tag fusion at the N-terminus (Supplementary Tables S1 and S2). Site-directed mutation in the sequence of DrXth in expression vector pET28a was generated with a QuickChange site-directed mutagenesis kit (Stratagene), and the oligonucleotide primers were shown in Supplementary Table S2. The constructed plasmid was transformed into *E. coli* BL21 (DE3), and the transformants were grown in the LB plate with 40 mg/L kanamycin.

DrXth, NΔ22DrXth, site-directed mutated DrXth, His-Strep-DrXth, and APE1 were expressed and purified in the same way. The expression strains were grown at 37°C in LB medium with 40 mg/L kanamycin to an optical density at 600 nm (OD600) of 0.6–0.8, and were induced at 30°C for 5 h by adding 0.2 mM isopropyl β-D-1-thiogalactopyranoside (IPTG). After harvesting, cells were re-suspended in buffer A [20 mM Tris-HCl (pH 7.5), 500 mM NaCl, 10% (v/v) glycerol, and 1 mM β-mercaptoethanol] consisting of the cOmplete Protease Inhibitor Cocktail (Roche Diagnostics, Switzerland) and lysed by sonication. The lysate was cleared by centrifugation at 15,000 *g* for 40 min at 4°C. Furthermore, the supernatant was loaded onto Ni-NTA column (AKTA pure 25, GE Healthcare, USA), using buffer A. Then, the target protein was eluted with elution buffer B [20 mM Tris-HCl (pH 7.5), 500 mM NaCl, 300 mM imidazole, and 10% (v/v) glycerol]. The fractions were desalted by loading onto desalt column (GE Healthcare, USA) with buffer C [20 mM Tris-HCl (pH 7.5), 150 mM NaCl, and 10% (v/v) glycerol]. After that, collected fractions were pooled and loaded onto HiTrap Q ion-exchange column (GE Healthcare). The target protein was eluted with buffer A by gradient elution. Finally, target protein was purified by a Superdex 200 (or 75) column (GE Healthcare) with buffer D [20 mM Tris-HCl (pH 7.5) and 150 mM NaCl]. DrPolA-C with N-terminal 6× His-tag was purified by Ni-NTA column, desalt column, Heparin HP column, and Superdex 200 column with buffer A, B, C and D. Expression and purification of DrPolX were performed as previously described (Leulliot et al., 2009). Fractions containing the protein were concentrated and stored at −80°C in 50% glycerol. The purified protein was verified by SDS-PAGE (Supplementary Figure S1).

### DNA Repair Assay

The oligonucleotides sequences used in this study with modifications and their complementary counterparts were purchased from Sangon and Eurogentec (Supplementary Table S2). The complementary oligonucleotides were annealed at 98°C for 10 min and gradually cooled down to the room temperature. For DrXth enzyme and the mutant enzymes nuclease activity assay, a certain concentration of DrXth protein and mutant proteins were mixed with 50 nM duplex oligonucleotide in a 10 μl reaction mixture containing 50 mM Tris (pH 7.5), 100 mM KCl, 5 mM MgCl_2_, 0.1 mg/ml bovine serum albumin (BSA), and 1 mM DL-Dithiothreitol (DTT) at 37°C for 5 min. To measure 3′-phosphate activity, 50 nM DNA substrates and enzymes were incubated with the reaction mixture containing 100 nM DrPolA-C enzyme, 50 mM Tris (pH 7.5), 100 mM KCl, 5 mM MgCl_2_, 100 μM dNTP, 0.1 mg/ml BSA, and 1 mM DTT at 37°C for 10 min. To measure NIR activity, we used 50 nM αdA duplex oligonucleotide to incubate with 5, 10, and 20 nM DrXth, and APE1 in the NIR reaction mixture consisted of 50 mM Tris (pH 6.8), 100 mM KCl, 1 mM MgCl_2_, 0.1 mg/ml BSA, and 1 mM DTT at 37°C for 10 min. For divalent metal ion preferences of the nuclease activity assay, DrXth (10 or 50 nM) and substrates (100 or 50 nM) were incubated with various concentration of MgCl_2_ or MnCl_2_ (1, 2, 5, and 10 mM) in the mixture consisted of 50 mM Tris (pH 7.5), 100 mM KCl, 0.1 mg/ml BSA, and 1 mM DTT 37°C for 5 min. The reaction mixture was stopped by adding 2× denaturing buffer (95% formamide and 50 mM EDTA) and heated at 98°C for 15 min, and then the samples were cooled down immediately to 4°C. The reaction products were separated by 15% polyacrylamide gels (7 M urea) in 1× TBE buffer. Gels were scanned by Typhoon FLA 9500 apparatus (GE Healthcare) and analyzed by ImageJ software.

For kinetic parameters analysis, 1–5,000 nM duplex oligonucleotide substrates were incubated with limited amount of DrXth (1–5 nM) in the standard reaction mixture (10 μl) according to the corresponding reaction described above at 37°C for 3 min. The reaction mixture was stopped by adding 2× denaturing buffer and heated at 98°C for 15 min, and then were cooled down immediately at 4°C. The reaction products were separated by 15% polyacrylamide gels (7 M urea) in 1× TBE buffer. The ratio of the reaction products vs. total substrates was calculated to obtain the enzyme reaction rate by ImageJ software. The data were fitted by the Michaelis-Menten equation in GraphPad Grism 5 in order to obtain the *K*_cat_ and *K*_m_.

For electrophoretic mobility shift assay (EMSA), the reaction mixture containing 50 mM Tris (pH 7.5), 100 mM KCl, and 0.1 mg/ml BSA were incubated with duplex oligonucleotides (50 nM) and DrXth or mutant proteins (50 or 100 nM) at 4°C for 30 min. Samples were separated on 15% native polyacrylamide gels in 1× TBE buffer. Gels were scanned by Typhoon FLA 9500 apparatus (GE Healthcare).

### Crystallization, Data Collection, and Structure Determination

The 22 amino acids N-terminal truncated DrXth protein was concentrated to 2.5 mg/ml and grown by sitting drop vapor diffusion method, using the Index^™^-HR-144 Scoring Sheet (Hampton Research, Aliso Viejo, CA, USA) to screen for DrXth crystals at 293 K. About 4 weeks later, DrXth crystals were obtained in the reservoir solution containing 0.1 M Bis-Tris PH 6.5, 20% w/v polyethylene glycol monomethyl ether 5000. Cryocooling was completed by soaking the crystals in the reservoir solution containing 20% glycerol for 3 min and flash freezing in liquid nitrogen. Diffraction intensities were recorded on beamline BL17U at Shanghai Synchrotron Radiation Facility (Shanghai, China) and were integrated and scaled using the XDS suite (Kabsch, 2010). The structure of DrXth was solved by molecular replacement using *N. Meningitidis* homolog [PDB: 2JC5; (Carpenter et al., 2007)] as the search model. The structure was refined by using PHENIX (Adams et al., 2010) and interspersed with the manual model building by using COOT (Emsley et al., 2010). All structural figures were rendered in PyMOL, and electrostatic surface potentials were calculated using APBS. The statistics of the data collection and refinement are list in Supplementary Table S3.

### DNA Damage Sensitivity Assay

*D. radiodurans* wild type R1, mutant, and complementary strains were cultivated with appropriate antibiotics in TGY media to OD_600_ = 1.0. Cells were washed and re-suspended in PBS solution. For γ-radiation treatment, cells were irradiated with ^60^Co gamma-ray (point source, Zhejiang Academy of Agricultural Sciences, Zhejiang, China) at several different doses (from 2 to 10 kGy) for 3 h. After irradiation, cells were serially diluted 1:10 with PBS solution and plated on TGY plates at 30°C for 3 days. For UV treatment, cells were firstly serially diluted 1:10 and plated on TGY plates. Then, the plates were exposed to UV lights at different doses (from 200 to 800 J/M^2^) and cultivated at 30°C for 3 days. For MMS treatment, cells were treated with different concentrations (from 10 to 30 mM) of MMS for 30 min. Then the cells were serially diluted 1:10 and plated on TGY agar plates. For H_2_O_2_ treatment, cells were incubated with H_2_O_2_ (ranged from 20 to 80 mM) for 30 min and then the reaction were stopped by excess catalase for 15 min. After that, the cells were serially diluted 1:10 and plated on TGY agar plates, and then cultivated at 30°C for 3 days. Three independent experiments were performed for each test. Survival fractions were determined by dividing number of colonies from the treated cells into those from the untreated control.

### Spontaneous Mutation Frequency Assay

To measure spontaneous mutation frequency, wild type, mutants, and complement cells were cultivated at early exponential phase, re-suspended, and plated on TGY agar plates with 50 μg/ml rifampicin at 30°C for 3 days (Kim et al., 2004). The spontaneous mutation rates were determined by dividing the total number of cells on TGY plates into that of colonies growing on TGY including rifampicin plates. Three independent replicates were performed for each strain.

### Pull-Down Assay

Pull-down assay were performed as previously described with some modifications (Cheng et al., 2017). 200 μl His-Strep-DrXth (0.5 mM) was incubated with 20 μl streptavidin beads at 4°C for 3 h and the beads were subsequently washed three times with wash buffer [20 mM Tris (pH 7.5), 100 mM NaCl, and 0.05% Tween 20]. Then, these beads were incubated with 400 μl PolX (0.5 mM) and the same amount of BSA as control at 4°C for 3 h. The beads were washed by wash buffer several times until the BSA was completely washed off. Finally, the beads were boiled and was analyzed by 12% SDS-PAGE.

## Results

### DrXth Is an ExoIII Family AP Endonuclease

**Figure 1:**
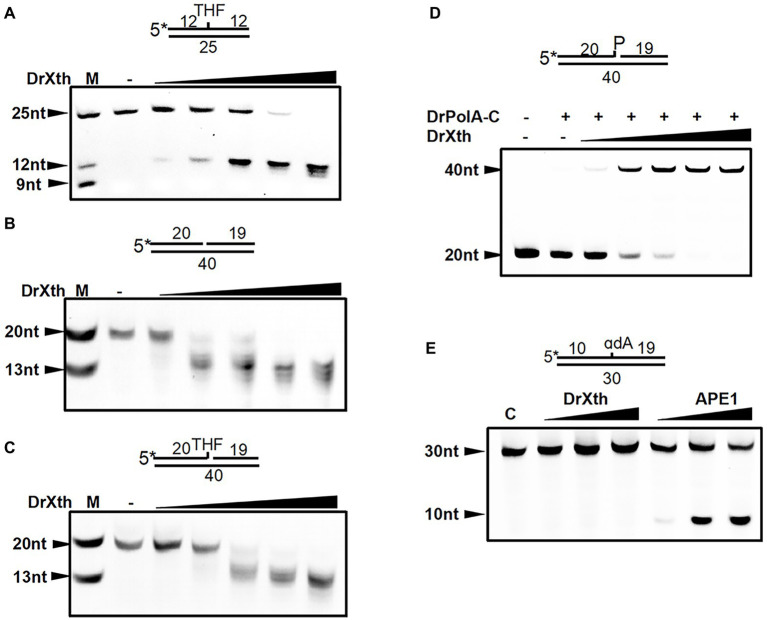
AP endonuclease from *Deinococcus radiodurans* (DrXth) possesses various enzyme activities with specific DNA substrates. **(A)** AP endonuclease activity. DNA substrates (50 nM) were incubated with DrXth (2, 5, 8, 10, and 20 nM) at 37°C for 5 min. **(B)** 3′–5′-exonuclease activity. **(C)** 3′-phosphodiesterase activity. DNA substrates (50 nM) were incubated with DrXth (20, 50, 80, 100, and 200 nM) at 37°C for 5 min **(B,C)**. **(D)** 3′-phosphatase activity. DrPolA-C (100 nM) was added to the reaction mixture containing DNA substrates (50 nM) and DrXth (20, 50, 80, 100, and 200 nM) at 37°C for 10 min. **(E)** Nucleotide incision repair (NIR) activity. DNA substrates (50 nM) was incubated with DrXth and APE1 (5, 10, and 20 nM) at 37°C for 10 min.

Sequence alignment suggested that *dr_0354* encodes a unique AP endonuclease (hereafter named DrXth) that is homologous to *E. coli* exonuclease III (Supplementary Figure S2). To confirm the biochemical activity of the DrXth protein, we first tested AP endonuclease activity using the 5′-FAM-labeled oligonucleotide duplex THF.T containing an abasic site in the middle of the DNA duplex. DrXth could incise the DNA substrate at the AP site, generating a 12 nucleotide (nt) product, and further digested the labeled DNA strand to shorter products with increasing enzyme concentrations (Figure 1A), suggesting that DrXth possesses both AP endonuclease and 3′–5′exonuclease activities. The 3′–5′-exonuclease activity was also confirmed using duplex DNA with a single nucleotide gap (Exo40.T) (Figure 1B). To examine the ability to eliminate the single-strand DNA break remnants with 3′-blocking sugar-phosphates, DrXth was incubated with a nicked DNA duplex containing 3′-THF (Exo40^THF^.T). Despite relatively low efficiency, DrXth acted on this substrate at high concentrations (Figure 1C). Moreover, previous studies showed that DNA polymerases such as PolA cannot extend DNA primer strands containing a 3′-phosphate (Exo40^P^.T) (Nakane et al., 2012). Interestingly, when DrXth was added to the DrPolA-C (the Klenow fragment of DrPolA with polymerase activity) reaction mixture with an Exo40^P^.T substrate, fully extended DNA products were observed, indicating that DrXth is able to process the 3′-phosphate terminus to generate DNA substrate for DrPolA-C (Figure 1D). To test whether DrXth can cleave the oxidized base in the NIR pathway independent of DNA glycosylases in the BER pathway, we tested α-2′-deoxyadenosine (αdA), a 5′-FAM-labeled DNA duplex with a damaged nucleotide. APE1 cleaved the 5′ side of the lesion site of the DNA duplex, but this product was not observed in the presence of DrXth (Figure 1E), suggesting that DrXth lacks NIR activity.

Steady-state kinetic parameters were measured to check the substrate specificity of DrXth (Table 1; Supplementary Figure S3). Among the tested DNA substrates shown in Figure 1, DrXth preferred to bind substrates containing AP sites (THF.T), as evidenced by the lowest *K*_m_ value. Notably, the overall efficiency (*K*_cat_/*K*_m_) of AP site substrates was 3.8- and 2.7-fold higher than that of nicked DNA duplexes with or without a 3′-phosphate (Exo40^P^.T and Exo40.T, respectively). Additionally, the nicked DNA duplex containing 3′-THF (Exo40^THF^.T) exhibited the lowest overall efficiency, indicating that DrXth might mainly function as an AP endonuclease *in vivo*.

**Table 1:** Steady state kinetic parameters of DrXth with different DNA substrates.

DNA substrate	*K*_m_, nM	*K*_cat_, min^−1^	*K*_cat_/*K*_m_, min^−1^·μM^−1^
THF.T	17.58 ± 1.93	4.00 ± 0.09	224.08
Exo40^THF^.T	35.18 ± 3.86	0.55 ± 0.01	15.63
Exo40^P^.T	74.93 ± 6.96	4.46 ± 0.09	59.52
Exo40.T	259.0 ± 32.95	21.89 ± 0.69	84.51

Each DNA substrate was used to measure a specific DNA repair activity under optimal reaction conditions (THF.T duplex for AP endonuclease activity; Exo40^THF^.T nicked DNA duplex containing 3′-THF for 3′-repair phosphodiesterase activity; Exo40^P^.T nicked DNA duplex containing 3′-P for 3′-phosphatase activity; Exo40.T recessed DNA duplex for the 3′–5′-exonuclease activity).

### Divalent Metal Ion Preferences of DrXth

**Figure 2:**
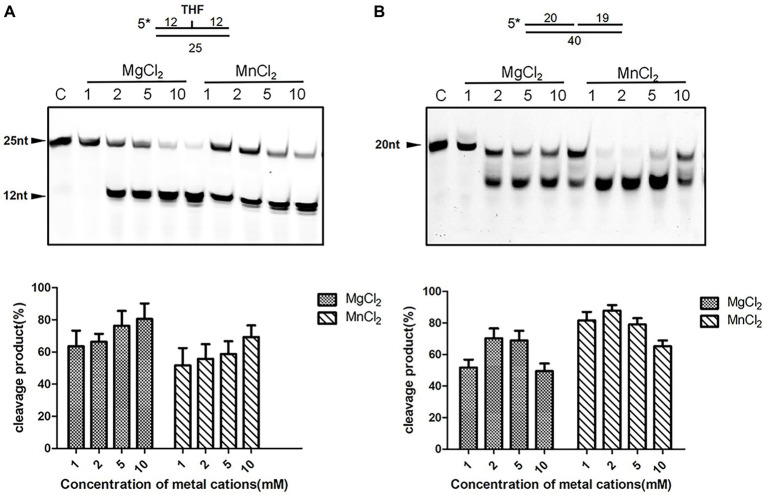
Divalent metal ion preference of the nuclease activity of DrXth. **(A)** Analysis of ion effects on AP endonuclease activity. One hundred nanomolar THF.T duplex was incubated with 2 nM DrXth in the presence of MgCl_2_ or MnCl_2_ (1, 2, 5, or 10 mM). **(B)** Analysis of ion effects on exonuclease activity. One hundred nanomolar 1 nt gap duplex DNA was incubated with 10 nM DrXth in the presence of MgCl_2_ or MnCl_2_ (1, 2, 5, or 10 mM). Reaction products were analyzed using ImageJ and cleavage percentage was calculated. Results were averaged from three independent experiments.

Divalent cations are indispensable for enzymatic activities of ExoIII family AP endonucleases. We therefore examined the metal ion preferences of DrXth by incubating with THF.T (to test AP endonuclease activity) and Exo40.T (to test 3′–5′ exonuclease activity) in the presence of Mg^2+^, Mn^2+^, Zn^2+^, or Ca^2+^. DrXth exhibited enhanced cleavage of the THF.T substrate with increasing concentrations (1 mM to 10 mM) of either Mg^2+^ or Mn^2+^ (Figure 2A), and the cleavage efficiency with Mg^2+^ was much higher than with Mn^2+^ (Figure 2A). In contrast, compared with Mg^2+^, elevated cleavage of Exo40.T was observed in the presence of Mn^2+^ (Figure 2B). However, high concentrations (10 mM) of Mg^2+^ and Mn^2+^ inhibited the cleavage of Exo40.T *in vitro*. Additionally, DrXth did not exhibit enzymatic activity against any of the tested substrates in the presence of Ca^2+^ or Zn^2+^ (Supplementary Figure S4). These results suggested that metal ions were necessary for DrXth enzyme activity, and Mg^2+^ and Mn^2+^ were preferred for endonuclease and exonuclease activities, respectively.

### Structure of DrXth and Comparison With ExoIII Family AP Endonucleases

**Figure 3:**
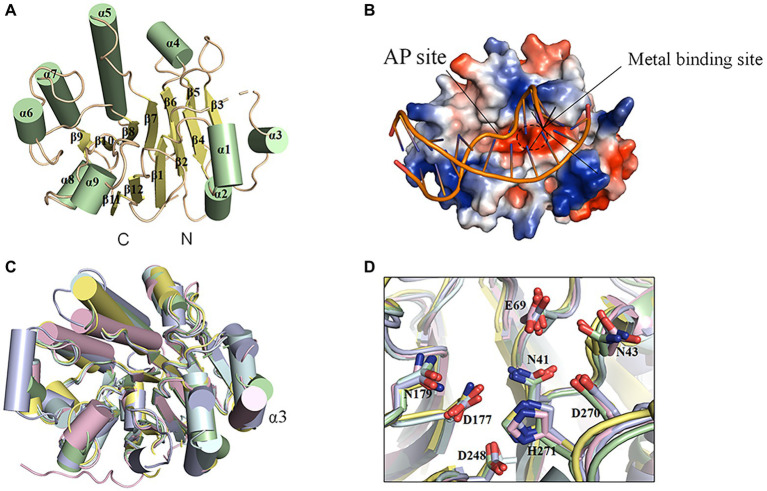
Structural features of DrXth and structural comparison among ExoIII family AP endonucleases. **(A)** Overall structure of DrXth. The α-helices and β-sheets are colored green and yellow, respectively. N and C represent the amino and carboxyl termini, respectively. **(B)** Distribution of the electrostatic surface of DrXth. Blue and red indicate negative and positive charge potentials at + and −70 kTe^−1^, respectively. DNA from the *Neisseria meningitidis* NApe-DNA complex was docked onto DrXth by superposition of DrXth and NApe. **(C)** Structural superimposition of DrXth (PDB: 6LPM, green) and NApe (PDB: 4B5H, yellow), APE1 (PDB: 1DEW, pink), Mth212 (PDB: 3FZI, cyan), and ExoIII (PDB: 1AKO, blue). **(D)** Comparison of key residues in the active site pocket.

The fully active, N-terminal truncated enzyme containing residues 23–283 (NΔ22DrXth) was purified and crystallized. The structure of NApe (PDB: 2JC5), AP endonuclease homolog in *Neisseria meningitides*, was used as a search model, and the crystal structure of DrXth was determined at 1.5 Å using the molecular replacement method (Supplementary Table S3). The overall structure of DrXth is composed of 12 β-sheets surrounded by 9 α-helices, forming a two-layer structure core (Figure 3A). Distribution of the electrostatic surface potential of DrXth revealed a central active site pocket with a negatively charged groove surrounded by a positively charged surface, which corresponds to divalent metal ion and DNA binding sites, respectively, as observed in the structures of other AP endonucleases (Figure 3B). The structure of DrXth is almost identical to that of other exonuclease type III AP endonucleases, namely *N. meningitides* NApe (PDB: 4B5H), human APE1 (PDB: 1DEW), *Methanobacterium thermoautotrophicum* Mth212 (PDB: 3FZI), and *E. coli* ExoIII (PDB: 1AKO), as shown in Figure 3C. However, different from these bacterial ExoIII type proteins, DrXth possessed the N-terminal region similar to APE1 (Supplementary Figure S2). The positively charged surface of DrXth appeared to be less extensive compared with other homologous proteins (Supplementary Figure S5). Additionally, the loop region connecting α3 and β3 displayed a noticeable deviation due to the lack of five residues between Pro80 and Gly81 (Figure 3C). The catalytic residues in the active site, namely Asn41, Asn43, Glu69, Asp177, Asn179, Asp248, Asp270, and His271, were superimposed with equivalent residues in the structures of other AP endonucleases (Figure 3D).

### Gly198 Is Critical for DNA Binding and Cleavage

**Figure 4:**
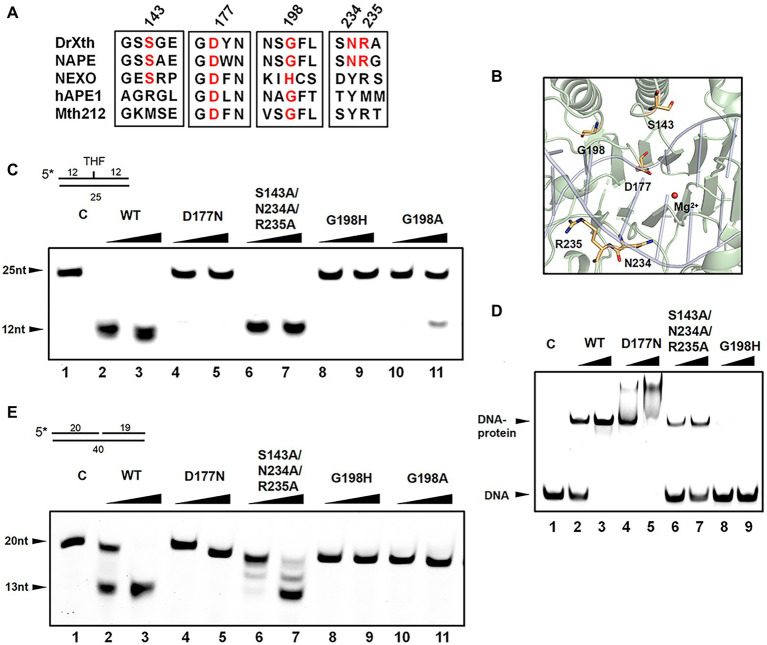
The roles of key amino acid residues in DrXth. **(A)** Sequence alignment of ExoIII family proteins DrXth (*D. radiodurans*), NApe (*N. meningitidis*), Mth212 (*Methanobacterium thermoautotrophicum*), APE1 (human), and NExo (*N. meningitidis*). Conserved amino acids are highlighted in red. **(B)** Key amino acids in DrXth. DNA and Mg^2+^ from the NApe-DNA complex were docked onto DrXth by superposition of DrXth and NApe. **(C)** AP endonuclease activity of DrXth and mutant proteins tested using the THF.T substrate. Fifty nanomolar DNA substrate was incubated with 50 or 100 nM DrXth and mutant proteins in reaction buffer at 37°C for 5 min. **(D)** Electrophoretic mobility shift assay (EMSA) of DrXth and mutant proteins using the THF.T substrate. 100 or 200 nM DrXth and mutant proteins were mixed with 50 nM THF.T substrate at 4°C for 30 min. **(E)** Exonuclease activity of DrXth and mutant proteins tested using the Exo40.T substrate. Fifty nanomolar DNA substrate was incubated with 50 or 100 nM DrXth and mutant proteins in reaction buffer at 37°C for 5 min.

Detailed sequence alignment and structural comparison led to the further mutagenesis studies to investigate the nuclease activities of DrXth (Figures 4A,B). Asp177 is highly conserved among AP endonucleases. In the NApe-DNA complex, this residue is involved in a hydrogen-bonding network in the active site, which plays a critical role in the hydrolysis reaction (Rothwell et al., 2000). As expected, THF.T substrate cleavage by the DrXth D177N mutant protein was severely inhibited (Figure 4C, lanes 4 and 5), even though D177N could bind to DNA containing an AP site (Figure 4D, lanes 4 and 5). A previous study identified the conserved X/S-Y/N/Q-R motif for DNA recognition in prokaryotes and X/R-Y-M/R in eukaryotes (Lu et al., 2012). Our results further confirmed that mutation of these three residues (S143A/N234A/R235A triple mutant) did not completely inhibit both AP endonuclease activity (Figure 4C, lanes 6 and 7) and exonuclease activity (Figure 4E, lanes 6 and 7), with only slightly decreased DNA binding capability compared with the wild-type protein (Figure 4D, lanes 6 and 7), which indicated that these residues were dispensable for DrXth nuclease activity.

It has been reported that the ExoIII family AP endonuclease NExo in *N. meningitides* lacks AP site cleavage activity due to sequence differences around residue His167 (Carpenter et al., 2007), for which the equivalent position in *D. radiodurans* is Gly198. Results of either EMSA and endonuclease assay revealed that mutation at this residue (G198H and G198A) drastically weakened the DNA binding and cleavage abilities of DrXth (Figure 4C, lanes 8–11; Figure 4D, lanes 8 and 9). Moreover, the exonuclease activities and exonuclease cleavage efficiencies of these mutants were severely impaired, similar to the AP endonuclease cleavage results (Figure 4E). These results indicated that residue Gly198 was critical for DNA substrate binding and cleavage by DrXth.

### DrXth Is Involved in DNA Damage Repair and Genome Stability *in vivo*

**Figure 5:**
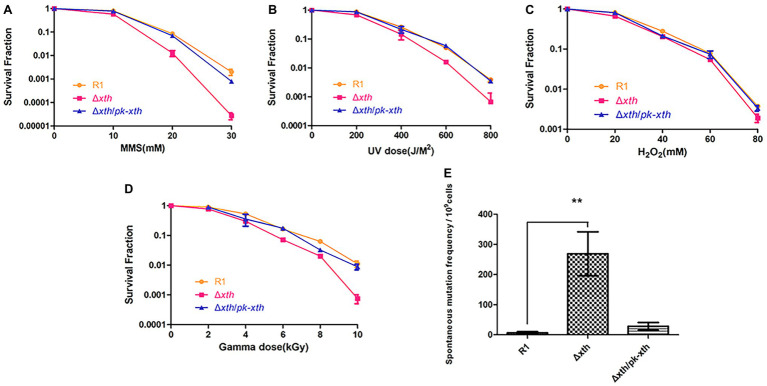
Stress resistance and spontaneous Rif^R^ mutation frequency of *D. radiodurans* strains. Survival rate of wild-type (R1), *drxth* deletion (*Δdrxth*) and, *drxth* complementation (*Δdrxth/pk-drxth*) strains following exposure to MMS **(A)**, UV radiation **(B)**, H_2_O_2_ **(C)** and gamma radiation **(D)** treatments. Cell viability was determined by comparing the number of colonies of treated cells with that of untreated cells incubated on TGY plates. **(E)** The spontaneous Rif^R^ mutation frequency of *D. radiodurans*. The spontaneous mutation rate is the number of colonies on TGY plates including rifampin divided by the total number of colonies on TGY plates. Average and standard deviations were calculated from three independent experiments. ^**^ *p* < 0.01.

One predominant type of DNA damage produced by radiation is the modification or loss of a base (Moeller et al., 2011). Methylated purines induced by methyl methanesulfonate (MMS) are excised by DNA glycosylases leading to the present of AP site (Ljungquist et al., 1976). Hydrogen peroxide (H_2_O_2_) oxidizes DNA bases and generates the single-strand DNA breaks with blocked 3′ termini (Demple et al., 1986). To explore the biological roles of *drxth*, we generated the knock-out strain Δ*drxth* and the complementary strain Δ*drxth/pk-drxth* (Supplementary Figure S6) and then measured their cell survival rate following exposure to MMS, UV radiation, and H_2_O_2_. As shown in Figure 5A, Δ*drxth* exhibited a dramatic decline in cell survival compared with the wild-type strain following MMS, while overexpression of *drxth* in Δdrxth/pk-*drxth* fully compensated for this phenotype. Similarly, Δ*drxth* showed sensitivity to various doses of UV radiation (Figure 5B), but the Δ*drxth* knock-out strain was not sensitive to H_2_O_2_ (Figure 5C). Due to the extreme resistance to gamma radiation of *D. radiodurans*, cell survival curves of R1, Δ*drxth*, and Δ*drxth*/*pk-drxth* strains were examined under γ-radiation, the *drxth* mutant strain was more sensitive to a high dose of γ-radiation than the R1 strain (Figure 5D).

It was reported that the spontaneous mutation frequencies of the *E. coli xthA* and *Helicobacter pylori xthA* mutant strains were significantly higher than those of the wild-type strains (Huang et al., 2006; Sikora et al., 2010). Herein, we used the Rif^R^/*rpoB* system to measure the spontaneous Rif^R^ mutation frequency of the *D. radiodurans* wild-type R1, *drxth* mutant, and complementary strains (Kim et al., 2004; Satoh et al., 2012). Notably, the *drxth* mutant strain exhibited a much higher (~40-fold) spontaneous mutation frequency than the R1 strain (Figure 5E). As expected, the spontaneous mutation frequency of the *drxth* complementary strain was compensated by overexpression of the DrXth protein (Figure 5E). These results further indicated that DrXth might play a crucial role in DNA damage repair pathways to maintain genome stability.

## Discussion

AP endonucleases with distinct activities play important roles in DNA repair and genome stability maintenance. A number of species possess more than one AP endonuclease. For instance, human cells contain two ExoIII family AP endonucleases, and *Bacillus subtilis* encodes two AP endonucleases (the EndoIV family enzyme BsNfo and the ExoIII family protein and ExoA) (Ibarra et al., 2008). Meanwhile, *Deinococcus geothermalis*, *Deinococcus deserti*, and *Deinococcus peraridilitoris* have two ExoIII family AP endonucleases (Lim et al., 2019). In the present study, we characterized the unique ExoIII family AP endonuclease DrXth from *D. radiodurans*, which is widely distributed in *Deinococcus* species. Whereas, *D. radiodurans* does not encode Nfo family AP endonucleases.

DrXth exhibited typical nuclease activities, including AP endonuclease, 3′-phosphodiesterase, 3′-phosphatase, and 3′–5′ exonuclease activities, but lacked NIR activity. It is reported that some AP endonucleases such as APE1 and Mth212 can initiate the NIR pathway (Ischenko and Saparbaev, 2002; Abeldenov et al., 2015). APE1 catalyzes the cleavage of AP- and 5,6-dihydrouridine (DHU)-containing substrates in the same active site (Felix and Silveira, 2011). Moreover, molecular dynamics simulations showed that the broad substrate specificity of APE1 is due to DNA distortion induced by the enzyme, which flips damaged nucleotides into the active site pocket (Kuznetsova et al., 2018). DrXth lacks NIR activity, similar to most prokaryotic ExoIII-like enzymes (e.g., EcXth and MtbXthA). However, the detailed mechanisms of NIR-deficient and NIR-proficient ExoIII family AP endonucleases have not yet been elucidated. The catalytic efficiency (*K*_cat_/*K*_m_) of human APE1 for AP site substrates is much higher than for nicked DNA, indicating that the incision efficiency is the major activity of AP endonucleases (Gelin et al., 2010). Similar to human APE1, DrXth yielded a higher *K*_cat_/*K*_m_ value with DNA substrates containing AP sites than other DNA substrates. Nevertheless, gap in *K*_cat_/*K*_m_ value of DrXth between each type of substrates was relatively smaller than APE1. Since this is the sole AP endonuclease in *D. radiodurans*, these catalytic efficiencies are comparable.

A previous study revealed that there is only one metal binding site in human APE1, and Mg^2+^ is required for both endonuclease and exonuclease activities (Lipton et al., 2008; Freudenthal et al., 2015). Under that same reaction conditions, a higher concentration of Mg^2+^ was required to reach the maximal endonuclease activity than exonuclease activity (Chou and Cheng, 2003). Depending on the concentration of Mg^2+^, the limiting stage of the process may change (Dyrkheeva et al., 2016). Our results confirmed that Mg^2+^ was preferred for the AP endonuclease activity of DrXth. However, our results showed that Mn^2+^ was preferred for the exonuclease activity of DrXth. It is known that *D. radiodurans* accumulates a high concentration of intracellular manganese (Daly et al., 2004). The detailed structural mechanisms about different metal ion preference and how these two activities coordinate require further investigation.

Previous studies showed that ExoIII family AP endonucleases usually adopt a four-layered α/β sandwich structure with a variable connecting loop. Comparison of ExoIII family AP endonucleases in eukaryotes and prokaryotes revealed minimal differences, with highly-conserved active sites and metal-binding residues. In DrXth, Asp177 strongly influences metal ion catalysis. However, in contrast with a previous proposal, the S143A/N234A/R235A triple mutant displayed a mild decrease in substrate binding ability and cleavage efficiency. Furthermore, our results suggested that Gly198 was the crucial residue involved in the distinct AP endonuclease and exonuclease activities of DrXth in *D. radiodurans*. Substitution of Gly by either His or Ala severely impaired the DNA binding and enzymatic activities. Based on the structure of DrXth, mutation of Gly198 to His or Ala was likely to prevent the enzyme from flipping out the abasic ribose (Supplementary Figure S7). The side chain of either His or Ala could affect the entrance of DNA substrates, indicating that a Gly residue was crucial for DrXth function.

The *drxth* mutant strain was sensitive to MMS, UV, and γ-radiation, suggesting that DrXth is involved in DNA repair pathways such as the BER pathway. However, knocking out *drxth* did not affect the survival of the bacterium following H_2_O_2_ treatment. Similarly, homozygous *Arabidopsis thaliana arp^−/−^* (AP endonuclease homologous gene) mutant exhibited no sensitivity to H_2_O_2_, but high sensitivity to *tert*-butyl hydroperoxide, suggesting that ARP is a major plant AP endonuclease that removes the special types of oxidative DNA base damage (Akishev et al., 2016). Meanwhile, the *D. radiodurans polX* mutant and endonuclease III (*nth*) mutants showed no significant difference from wild type in H_2_O_2_ stress resistance (Khairnar and Misra, 2009; Hua et al., 2012). It has also been shown that mutation of both *ttendolVI* and *ttpolX* resulted in a significant decline in activity following exposure to H_2_O_2_ in *Thermus thermophilus* (Nakane et al., 2012). Indeed, the *D. radiodurans* Δ*polX*Δ*xth* double mutant displayed significantly reduced resistance to H_2_O_2_ (Supplementary Figure S8). In addition, a recent research revealed that DrEndoIII2 contributes to the major EndoIII enzyme activity and DrEndoIII1 might be responsible for the repair and processing for a part of oxidized bases and coordination with DrXth (Sarre et al., 2019), which may play an alternative role in the *drxth* knock-out strain under oxidative stress. These results imply that PolX and DNA glycosylase may play a backup role for DrXth, and cooperate and coordinate in the BER repair pathway.

As an essential enzyme in the BER pathway, AP endonuclease is able to interact with upstream and downstream BER proteins. It was reported that APE1 stimulates the activity of DNA glycosylase OGG1 and strengthens its specificity (Sidorenko et al., 2007). In addition, APE1 in concert with Polβ generates an APE1-DNA-Polβ complex that stimulates lyase activity and the ability of Polβ to fill the gaps in nicked substrates (Liu et al., 2007). APE1 N-terminal domain (33 amino acids) are required to interact with many interaction partners and promote the coordination of total AP site repair (Roychoudhury et al., 2017). However, whether the N-terminal region of DrXth has the similar function to that of APE1 needs further study. Moreover, our results have confirmed the interaction between DrXth and DrPolX (Supplementary Figure S9), indicating that DrXth may be important in the central stages of the BER pathway by coordinating with upstream and downstream enzymes. Taken together, our findings provide new insights into the properties and role of the single AP endonuclease DrXth in this extremophile under DNA damage stress.

## DatA Availability Statement

The coordinates and structure factors have been deposited to Protein Data Bank with accession codes 6LPM.

## Author Contributions

YHu conceived the project. YHe, LW, and YHu were responsible for the experimental design and drafted the manuscript. YW, KC, CQ, and YX constructed the vector and mutants and purified the proteins. YHe and YW performed the enzymatic activity assay and phenotypic assay. YZ determined and analyzed the structure. YHe, BT, and HX took part in data analysis. All authors reviewed the manuscript and approved the version to be published.

## Conflict of Interest

The authors declare that the research was conducted in the absence of any commercial or financial relationships that could be construed as a potential conflict of interest.

## Acknowledgments

We would like to thank the staffs at the Shanghai Synchrotron Radiation Facility (SSRF in China) for assistance in data collection.

## Supplementary Material

The Supplementary Material for this article can be found online at:

https://www.frontiersin.org/articles/10.3389/fmicb.2020.01178/full#supplementary-material
