# Supplementary material for: Structural and Functional Characterization of a Unique AP Endonuclease From Deinococcus radiodurans
Source: Front Microbiol. 2020 Jun 5;11:1178. doi: 10.3389/fmicb.2020.01178 (PMC7548837; doi:10.3389/fmicb.2020.01178)
Supplement: Supplementary file 1 [file Data_Sheet_1.pdf]

Supporting information

Structural and functional characterization of an AP endonuclease from *Deinococcus radiodurans*

Supplementary Table1. Data collection, phasing and refinement statistics

Supplementary Table2. Strains and plasmids used in this study

Supplementary Table3. Primes and oligonucleotides used in this study

Supplementary Figure1. Sequence alignments among DrXth and other representative ExoIII family AP endonucleases

Supplementary Figure2. Kinetic of DrXth nuclease activity

Supplementary Figure3. Analysis of ion effects on AP endonuclease activity

Supplementary Figure4. Structural comparison among DrXth, NApe, ExoIII and APE1

Supplementary Figure5. Deletion of *drxth* gene in D.radiodurans R1 strain

Supplementary Figure6. Predicted structure of Gly198 mutant DrXth

Supplementary Figure7. Phenotypes of R1 strain and mutant strain under H_2O_2 stress

Supplementary Figure8. Interaction assay between DrXth and DrPolX

Supplementary Figure9. SDS-PAGE analysis of the purified full-length type and N-terminal truncated DrXth

Supplementary Table1. Data collection, phasing and refinement statistics

	DrXth
**Data collection**	
Space group	$P2_12_12_1$
Cell dimensions	
a, *b*, *c* (Å)	57.42<br>58.86<br>74.28
Wavelength (Å)	0.9792
Resolution (Å)	30.0-1.50
R_{sym} (%)	6.6 (47.3)
$I/\sigma I$	14.6 (4.3)
Completeness (%)	98.2<br>(91.0)
Redundancy	5.1 (3.4)
**Refinement**	
Resolution (Å)	30.0-1.50
No. reflections	40705
$R_{work/}$ R_{free}	18.6/20.4
No. atoms	
Protein	1941
Water	243
B-factors	
Protein	19.3
Water	32.0
R.m.s deviations	
Bond lengths (Å)	0.005
Bond angles (°)	0.865

*Highest resolution shell is shown in parenthesis.

Supplementary Table2. Strains and plasmids used in this study

**Strain and plasmid**	**Description**	**Reference and source**
**strains**		
Deinococcus radiodurans		
R1	ATCC 13939	Laboratory Stock
Δxth	R1 but *xth::kan*	This study
Δxth/pk-xth	*Δxth* but pRADK::*xth*	This study
Escherichia coli		
DH5α	*E. coli* cloning strain	TransGen
BL21(DE3)	*E. coli* expression strain	TransGen
**plasmids**		

pET28a	T7 promoter, T7 transcription start, His•Tag coding sequence, T7•Tag coding sequence, Multiple cloning sites(BamH I -Xho I), T7 terminator, *lacI*coding sequence, pBR322 origin, *Kanr*, f1 origin, 6His-tag coding sequence	Novagen
pET28S	pET28 plasmid modified with a Strep-tag sequence (WSHPQFEK)	Laboratory Stock
pET28aXth	pET28a containing wild type *xth* gene	This study
pET28aXthΔ22	pET28a containing wild type N-terminus domain of *xth* gene	This study
pET28S-Xth	pET28S containing wild type *xth* gene	
pET28aD177N	pET28a containing *xth* directed site D155N mutation gene	This study
pET28a S143A/N234A/R235A	pET28a containing *xth* directed site S121A/N212A/R213A triple mutation gene	This study
pET28aG198H	pET28a containing *xth* directed site G176H mutation gene	This study
pET28aG198A	pET28a containing *xth* directed site G176A mutation gene	This study
pET28aAPE	pET28a containing *ape* gene	This study
pET28aPolA-C	pET28a containing C-terminus domain of *drpolA* gene	This study
pET28aPolX	pET28a containing *polx* gene	Laboratory Stock
PRADK	*D. radiodurans* shuttle vector	Laboratory Stock
pk-xth	pRADK::*xth*	This study

Supplementary Table3. Primes and oligonucleotides used in this study

**Prime**	**Sequence(5’-3’)**
**construction and complement of drxth mutant**	
xth p1	CCCCCGAACTCGACGTG
xth p2(HindIII)	CCCAAGCTTCGGCGCCGACCATAGC
xth p3(BamHI)	CGGGATCCACCCTACCTTCTCCCCAGTACC
xth p4	TCTGCGCTGTCCTCGGTG
xth p5	TGCTTCTGCAAGAAGTCCGC
xth p6	GATTCCAGCTCCACCCACCC
**Expression of proteins**	
Xth F (NdeI)	GGAATTCCATATGTTGAGCCTCCTTGCCCCA
Xth R (BamHI)	CGGGATCCTCATTCAGATTCCAGCTCCACC
XthΔ22 (NdeI)	GGAATTCCATATGATGTCTGCCCCCGCCG

XthΔ22 (BamHI)	CGGGATCCTCATTCAGATTCCAGCTCCACC
APE1F(NdeI)	GGAATTCCATATGATGCCGAAGCGTGGGAAA
APE1R(BamHI)	CGGGATCCTCACAGTGCTAGGTATAGGGTGATAGG
PolA-C(NdeI)	GGAATTCCATATGATGGGGCTGAACGGGCCA
PolA-C (BamHI)	CGGGATCCTCACTTCGTGTCAAACCAGTTCG
**Site-directed mutagenesis**	
xth G198H(F)	GCTCGTGGGGCAGGAAATGGCTGTTTTTCTGGTTGC
xth G198H(R)	GCAACCAGAAAAACAGCCATTTCCTGCCCCACGAGC
Xth D177N(F)	GGCGATGTTGTAGTTGCCGCCGATGACGA
Xth D177N(R)	TCGTCATCGGCGGCAACTACAACATCGCC
XTH G198A(F)	CGTGGGGCAGGAAAGCGCTGTTTTTCTGG
XTH G198A(R)	CCAGAAAAACAGCGCTTTCCTGCCCCACG
Xth S143A(F)	CGCCTCGCCGGCGCTGCCGCTCG
Xth S143A(R)	CGAGCGGCAGCGCCGGCGAGGCG
Xth N234R235A(F)	GGCGTAGGCATTGGCGGCGGCGCTCCACCAGGTGTAC
Xth N234R235A(R)	GTACACCTGGTGGAGCGCCGCCGCCAATGCCTACGCC

Oligonucleotides for enzyme activity assay

Type of substrate	Sequence	label
THF.T	5′*GCTATGGACTAAFAATGACTGCGTG 3′	F(THF)
	3′CGATCCTGATTTTTACTGACGCAC5′	*(FAM)
Exo40.T	5′*ATGACAACTAAAGCAACACC3′ 5′GATAGAACGACCGCCAGTG3′	*(FAM)
	3′TACTGTTGATTTCGTTGTGGTCTATCTTGCTGGCGGTCAC5′	
Exo40^{P}.T	5′*ATGACAACTAAAGCAACACC^{P}3′ 5′GATAGAACGACCGCCAGTG3′	*(FAM)
	3′TACTGTTGATTTCGTTGTGGTCTATCTTGCTGGCGGTCAC5′	
Exo40^{THF}.T	5′*ATGACAACTAAAGCAACACCF 5′GATAGAACGACCGCCAGTG3′	F(THF)
	3′TACTGTTGATTTCGTTGTGGTCTATCTTGCTGGCGGTCAC5′	*(FAM)
αdA	5′*TGACTGCATAXGCATGTAGACGATGTGCAT3′	X(αdA)
	3′ACTGACGTATACGTACATCTGCTACACGT5′	*(FAM)

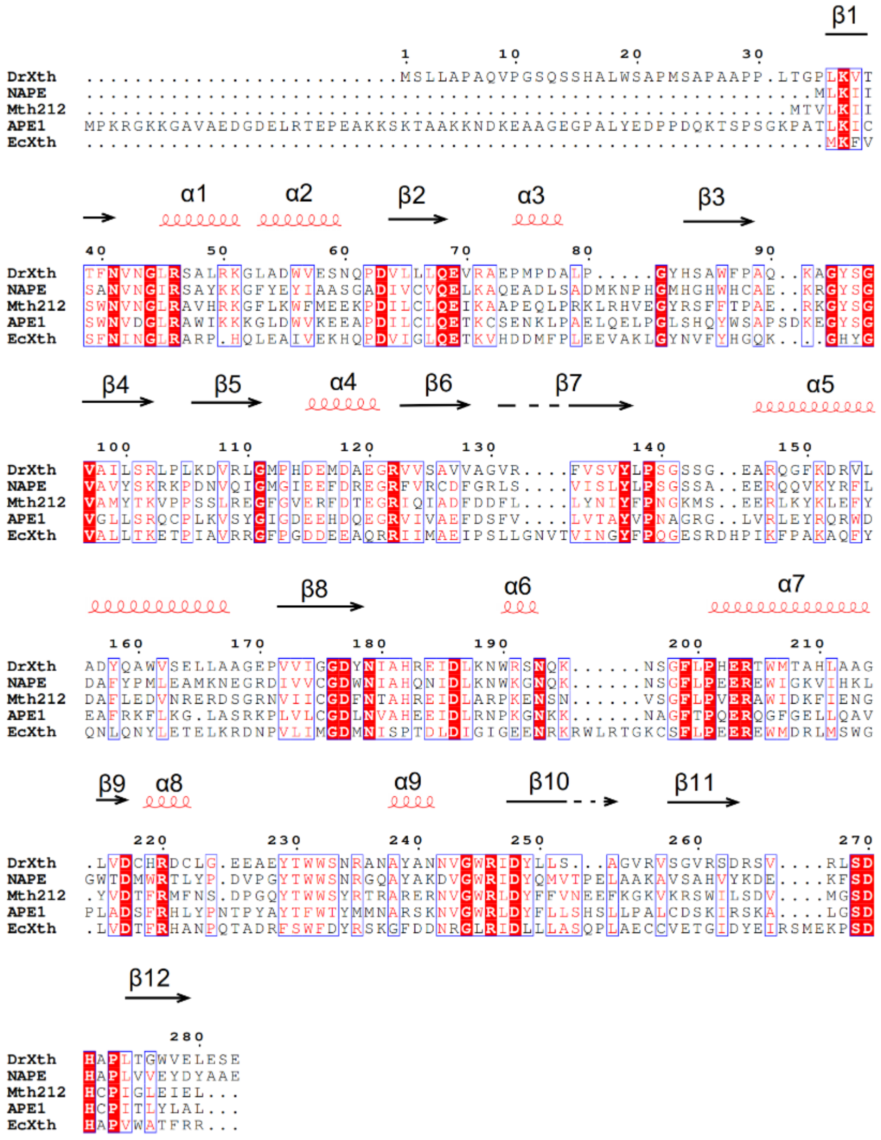

Supplementary Figure1. Sequence alignments among DrXth and other representative ExoIII family AP endonucleases. DrXth, *Deinococcus radiodurans*; NApe, *Neisseria meningitidis*; Mth212, *Methanobacterium thermoautotrophicum*; APE1, *Homo sapiens*; EcXth, *Escherichia coli*.

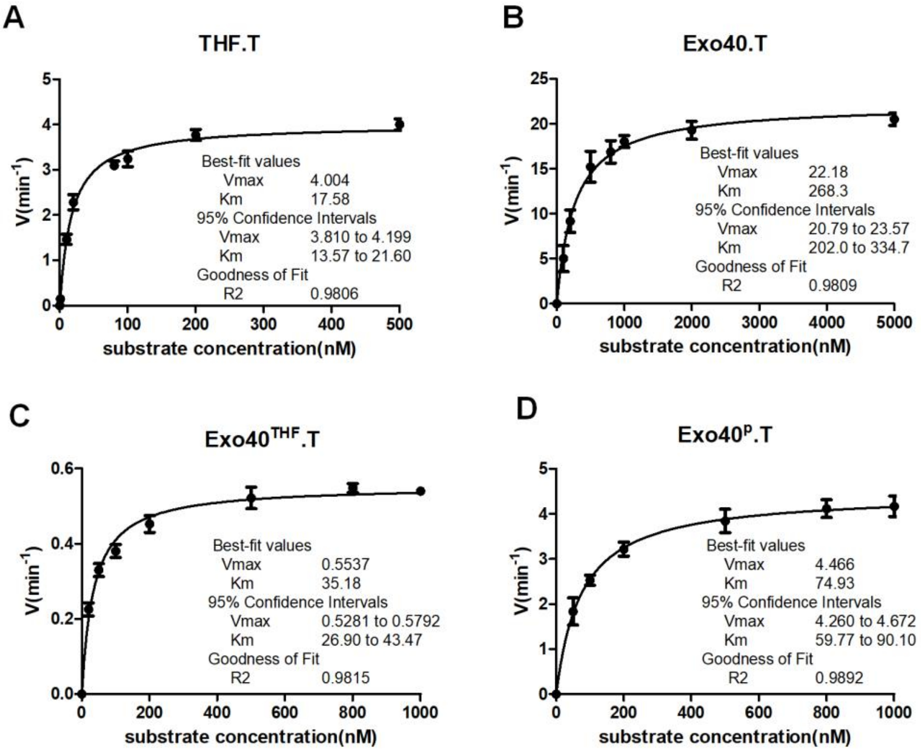

Supplementary Figure2. Kinetic of DrXth nuclease activity. (A) DrXth (1 nM) was incubated with increasing amounts of THF.T substrate (1, 10, 50, 80, 100, 200, 500 nM) at 37°C for 3 min. (B) DrXth (3 nM) was incubated with increasing amounts of Exo40.T substrate (200, 500, 800, 1000, 2000, 5000 nM) at 37°C for 3 min. (C) DrXth (3 nM) was incubated with increasing amounts of Exo40THF.T substrate (20, 50, 100, 200, 500, 800, 1000 nM) at 37°C for 3 min. (D) DrXth (3 nM) was incubated with increasing amounts of Exo40P.T substrate (20, 50, 100, 200, 500, 800, 1000 nM) at 37°C for 3 min. The data were fitted by the Michaelis–Menten equation in GraphPad Grism 5 in order to obtain the Kcat and Km. Best-fit values, 95% confidence intervals and goodness of fit are shown.

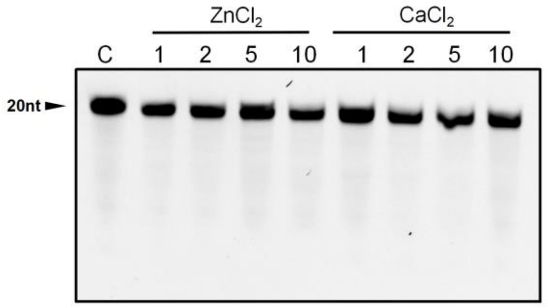

Supplementary Figure3. Analysis of ion effects on AP endonuclease activity. 100 nM THF.T duplex was incubated with 2 nM DrXth in the presence of $CaCl_2$ or $ZnCl_2$ (1, 2, 5 or 10 mM) at 37°C for 5 min.

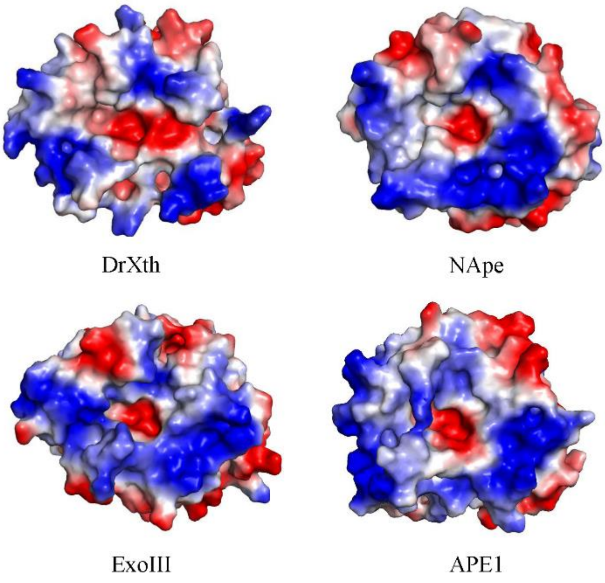

Supplementary Figure4. Structural comparison among DrXth, NApe, ExoIII and APE1. The structure shows the distribution of the electrostatic surface. Blue and red represent negative and positive charge potencial at + and − 70 kTe^{-1}scale, respectively.

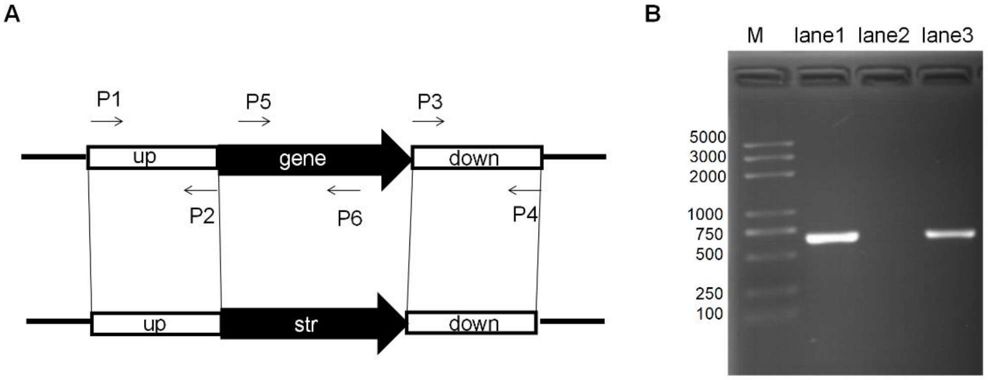

Supplementary Figure5. Deletion of *drxth* gene in D.radiodurans R1 strain (A) Scheme of gene mutation by homologous recombination that replaced the target ORFs with streptomycin-resistant fragment. P1, P2, P3, P4, P5 and P6 refer to the primer pairs (Supporting information Table3). (B) PCR analysis to confirm the mutation of dr0354. An interior DNA fragment (652 bp) of the targeted gene was detected by amplification using primers P5 and P6. No products corresponding to the size of the fragment was observed from mutant (lane 2) but observed in the wild type (lane 1) and compensation type (lane 3), suggesting that the wild type alleles were completely replaced by streptomycin-resistance fragment in the mutant.

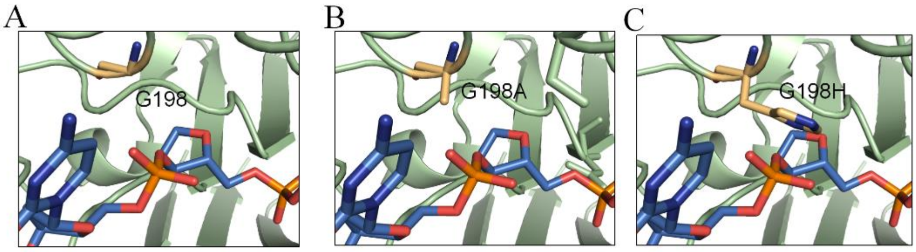

Supplementary Figure6. Predicted structure of Gly198 mutant DrXth. DNA from NApe-DNA complex were docked onto the DrXth by superposition between DrXth and NApe. (A) DrXth (B) Substitue Ala for Gly198 (C) Substitute His for Gly198.

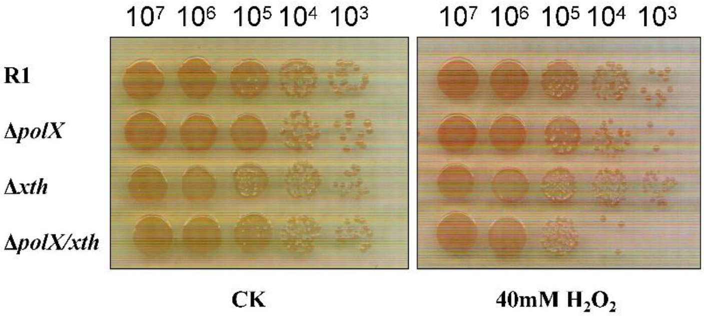

Supplementary Figure7. Phenotypes of R1 strain and mutant strain under H_2O_2 stress. Growth and H_2O_2 resistance features of wild type (R1), *drpolX* disruptant (Δ*polX*), *drxth* disruptant (Δ*xth*), *drpolX*-*drxth* double disruptant (Δ*polX*/ *xth*). Cells were incubated with H_2O_2 (40 mM) for 30min and then the reaction were stopped by excess catalyase for 15 min. After treatment, the cells were serially diluted 1:10 and spotted on TGY agar plates, and then cultivated at 30°C for 3 days.

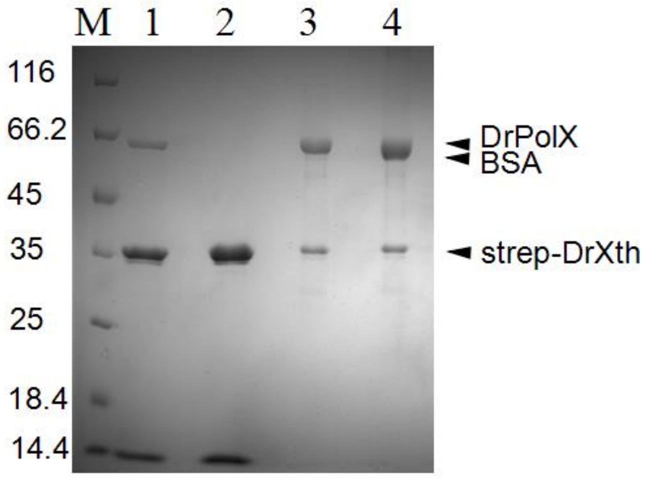

Supplementary Figure8. Interaction assay between His-Strep- DrXth and DrPolX. His-Strep-DrXth (N-terminal fused streptavidin tag) binding with strep-beads were rotating incubated with DrPolX and BSA at 4 ℃ for 3h. Lane1: His-Strep-DrXth and DrPolX were pulled-down by strep-beads. Lane2: His-Strep-DrXth and BSA (input control) were pulled by strep-beads. Lane3: His-Strep-DrXth and DrPolX. Lane4: His-Strep-DrXth and BSA (input control).

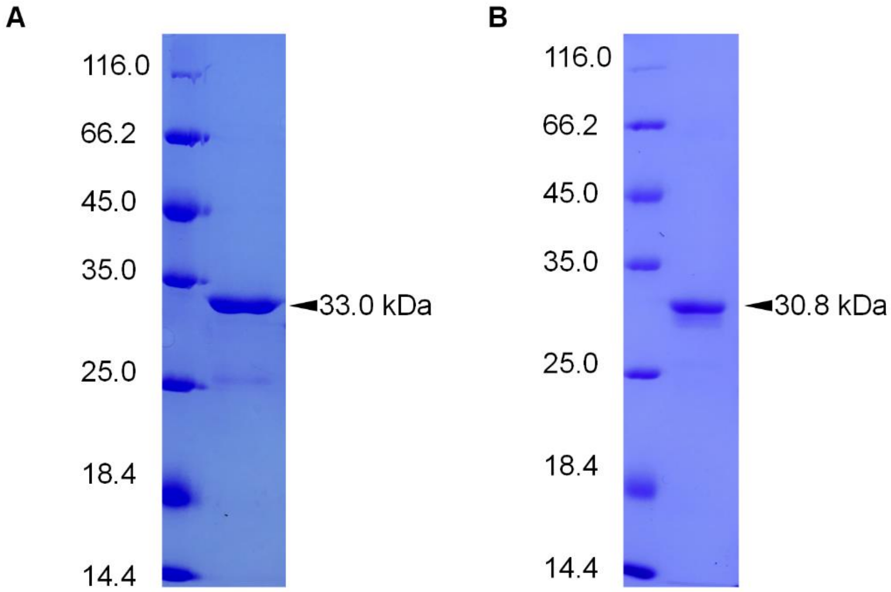

Supplementary Figure9. SDS-PAGE analysis of the purified full-length type and N-terminal truncated DrXth. (A) Molecular weight of full-length type DrXth protein with His-tag is 33.0 kDa. (B) Molecular weight of N-terminal truncated DrXth protein (NΔ22DrXth) with His-tag is 30.8 kDa.
